# Examining existing strategies to prevent multimorbidity – a scoping review

**DOI:** 10.1093/eurpub/ckac129.290

**Published:** 2022-10-25

**Authors:** K Nicholson, T Makovski, M van den Akker, S Stranges

**Affiliations:** 1 Department of Epidemiology and Biostatistics, Western University, London, Canada; 2 Department of Epidemiology, Maastricht University, Maastricht, Netherlands; 3 Institute of General Practice, Goethe University, Frankfurt am Main, Germany; 4 Department of Precision Health, Luxembourg Institute of Health, Strassen, Luxembourg

## Abstract

Multimorbidity has been acknowledged as the “defining challenge” for health systems around the world, but current research and policy are primarily focused on the management of multimorbidity after it occurs or the prevention of adverse events in those individuals who are already living with multimorbidity. There is still a significant need to develop and establish effective and equitable primary prevention strategies in order to avoid the occurrence of multimorbidity across populations. This scoping review aims to identify existing strategies, programs and policies that are focused on the prevention of multimorbidity across various settings and countries around the world. To identify relevant publications, two databases will be searched: PubMed and Embase. The search strategies will be adjusted for each database and will include variations of the keywords multimorbidity, prevention and strategy. This review will specifically include publications that are original research, focused on multimorbidity and published in English. However, there will be no restrictions on the location of the research or the age of the target sample or population. For the full text screening phase, more specific criteria will apply and information will be extracted from the final set of retained studies. This information will be summarized and will include key factors such as study design, study setting, type of prevention programs, scope of programs (e.g. national, regional, local, etc.) and target population (e.g. age groups, socioeconomic groups, etc.). This scoping review will describe existing prevention programs and highlight areas of gaps and opportunities in the prevention of multimorbidity.

